# DNA Extraction with DNAzol and LAMP, Performed in a Heating Block as a Simple Procedure for Detection of *Mycobacterium tuberculosis* in Sputum Specimens

**DOI:** 10.3390/mps1040037

**Published:** 2018-10-23

**Authors:** Álvaro Rodríguez-García, Rosa E. Mares, Patricia L. A. Muñoz, Samuel G. Meléndez-López, Alexei F. Licea-Navarro, Marco A. Ramos

**Affiliations:** 1 Facultad de Ciencias Químicas e Ingeniería, Universidad Autónoma de Baja California, Calzada Universidad 14418, Tijuana, BCN 22390, Mexico; alvaro.rodriguez@uabc.edu.mx (Á.R.-G.); rmares@uabc.edu.mx (R.E.M.); lilian.munoz@uabc.edu.mx (P.L.A.M.); samuelmelendez@uabc.edu.mx (S.G.M.-L.); 2 Laboratorio de Diagnóstico Clínico, Hospital General de Tijuana, Vía de la Juventud Oriente 4910, Tijuana, BCN 22010, Mexico; 3 Departamento de Innovación Biomédica, Centro de Investigación Científica y Educación Superior de Ensenada, Carretera Ensenada-Tijuana 3918, Ensenada, BCN 22860, Mexico; alicea@cicese.mx

**Keywords:** tuberculosis, molecular detection, loop-mediated isothermal amplification

## Abstract

Tuberculosis (TB) remains as a major public health issue in developing countries. Accurate detection is essential for the proper management of patients with active disease. Here, we present a simple DNAzol-LAMP (loop-mediated isothermal amplification) procedure for the detection of *Mycobacterium tuberculosis* in sputum specimens. Twenty smear-positive sputum samples were analyzed as follows: (i) Genetic material was extracted by a standard DNAzol protocol, and (ii) mycobacterial DNA was detected by a typical TB-specific loop-mediated isothermal amplification method. Results and diagnostic test performance attests to the suitability of the proposed procedure.

## 1. Introduction

Tuberculosis (TB) continues among the major threats to public health. Worldwide, morbidity, and mortality rates indicate that eradication remains out of reach [1]. In 2015, the WHO estimated 10.4 million new cases [2], suggesting that both late detection and misdiagnosis sustain the disease prevalence [3]. Two core methods, smear microscopy, and bacterial culture, are the foremost means for the detection of *Mycobacterium tuberculosis*, the leading causative agent of TB in humans [4]. Smear microscopy is inexpensive, but has low sensitivity, while bacterial culture (the gold standard) is sensitive, but it takes up to six weeks to provide a reliable result. Despite their intrinsic disadvantages, to date, these methods are applied for routine mycobacteriology [5,6].

The development of accurate diagnostic systems is essential to reducing TB incidence [4]. Several nucleic acid amplification techniques (NAAT), such as polymerase chain reaction (PCR) methods have addressed this demand, and have proven their diagnostic value. Even so, the requirement of expensive equipment (i.e., thermal cyclers) restricts their inclusion as a routine method in clinical laboratories with budget restrictions [4,5,6,7]. The loop-mediated isothermal amplification (LAMP) method, an alternate NAAT, allows for the use of a heating block to carry out the reaction, conceding a simple adaptation to settings with limited resources [8,9]. Since LAMP has shown reliability for TB detection [10], the WHO Expert Group agreed that it has outstanding potential as a rapid diagnostic tool [11].

In 2015, Mexico reported 21 cases per 100,000 people [2], indicating that TB remains prevalent in the current population. Remarkably, the Northern States listed the highest incidence values, with Baja California at the forefront [12,13]. Here, we present a simple DNAzol–LAMP procedure for the detection of *M. tuberculosis* in sputum specimens. Also, endpoint PCR and bacterial culture results were available for comparison purposes, and the analysis of diagnostic test performance.

## 2. Materials and Methods

The study was conducted in accordance with the Declaration of Helsinki, and the protocol was approved by the Ethics Committee of the General Hospital of Tijuana (Project identification code 10/05/2016). No consent for participation was required. Sputum samples, DNA extracts, and bacterial cultures were analyzed anonymously. No human tissue or any other biological materials were used.

### 2.1. Materials

The BBL MycoPrep specimen digestion/decontamination kit was supplied by Becton, Dickinson, and Company (Sparks, MD, USA). The DNAzol BD reagent was acquired from Invitrogen (Waltham, MA, USA). *Bst* 2.0 DNA polymerase was available from New England Biolabs (Ipswich, MA, USA), while Taq DNA polymerase were from Qiagen (Germantown, MD, USA). DNA analysis reagents were provided by Bio-Rad Laboratories (Hercules, CA, USA). Unless otherwise mentioned, additional reagents were obtained from Sigma-Aldrich (St. Louis, MO, USA). Synthetic oligonucleotides, based on the primers designed to target the IS6110 sequence [10], were obtained from Eurofins MWG (Louisville, KY, USA). All materials were of biochemical or biotechnological research grade.

### 2.2. Sputum Specimens

Sputum specimens were provided by patients suspected of having TB disease and collected by the personnel of the TB Diagnostics Lab at the General Hospital of Tijuana (GHT). Digestion-decontamination was completed using the BBL MycoPrep routine. For this study, 20 sputum samples positive for acid-fast bacilli (i.e., smear-positive) were analyzed. Sample handling and other subsequent procedures were performed in accordance with protocols approved by the Institutional Ethics Review Board (GHT).

### 2.3. Extraction of DNA from Sputum Samples

Genetic material was extracted using the DNAzol BD reagent as recommended. Briefly, 0.2 mL sediment suspension was mixed with 0.5 mL of 1× Dulbecco’s PBS solution. Bacterial lysis was achieved heating at 80 °C for 10 min and cooling for 1 min on ice. One milliliter of DNAzol was added and mixed thoroughly. Cell debris was removed by centrifugation at 10,000× *g* for 10 min. The supernatant was mixed with 0.5 mL of cold ethanol and centrifuged at 14,000× *g* for 10 min. After washing, the precipitated DNA was dissolved in 30 μL of 8 mM NaOH.

### 2.4. Typical LAMP Assay

Reactions (25 μL) were completed in 1× isothermal buffer containing 1.6 μM FIP/BIP (forward/backward inner primers), 0.2 μM FOP/BOP (forward/backward outer primers), 0.8 μM FLP/BLP (forward/backward loop primers), 0.8 M betaine, 2 mM deoxynucleotide (dNTP) solution mix, 4 mM MgSO_4_, 8 units *Bst* 2.0 DNA polymerase, and 1 μL template [10]. Isothermal conditions were 90 min at 63 °C, and 5 min at 90 °C. All reactions were performed using a VWR Digital Dry Block Heater (i.e., heating block). Loss of volume was avoided by supplementing the reaction tube with 25 μL of mineral oil.

### 2.5. Standard Endpoint PCR Assay

Reactions (20 μL) were achieved in 1× Taq PCR Master Mix containing 1 μM FOP/BOP and 1 μL template. Endpoint amplifications were performed in a LabNet Multigene Thermal Cycler with the following conditions: 2 min at 94 °C; 45 cycles of exponential amplification (20 s at 94 °C, 20 s at 55 °C, 20 s at 72 °C); and 7 min at 72 °C.

### 2.6. Analysis of Amplification Products

Amplification products were separated by agarose gel electrophoresis, stained with ethidium bromide, visualized under UV light, and documented using a Bio-Rad GelDoc EZ System. Also, LAMP products were visually-analyzed using hydroxy naphthol blue dye as a chromogenic indicator [14]. However, judging positive (blue) or negative (violet) represented an experimental endeavor, due to color resemblance; hence this approach was not considered.

### 2.7. Assay Settings

Reference DNA was used as a template to validate both LAMP and PCR methods, and it served as the positive control throughout routine assays of sputum samples. Regularly, a reaction lacking a template was regarded as the negative control (i.e., blank reaction). Cross-contamination was prevented by using separate areas for routine work, such as the extraction of DNA and the preparation of responses. Samples were analyzed by duplication when a doubtful outcome was obtained.

### 2.8. Statistical Analysis of Data

Degrees of agreement between assays was pondered by kappa (*κ*) statistics using the GraphPad module for quick calculations [15]. Diagnostic performance: sensitivity, specificity, and predictive values, were calculated using the OpenEpi module for diagnostic test evaluation [16].

## 3. Results and Discussion

### 3.1. Assessment of the LAMP Assay Performed in a Heating Block

**Figure 1:**
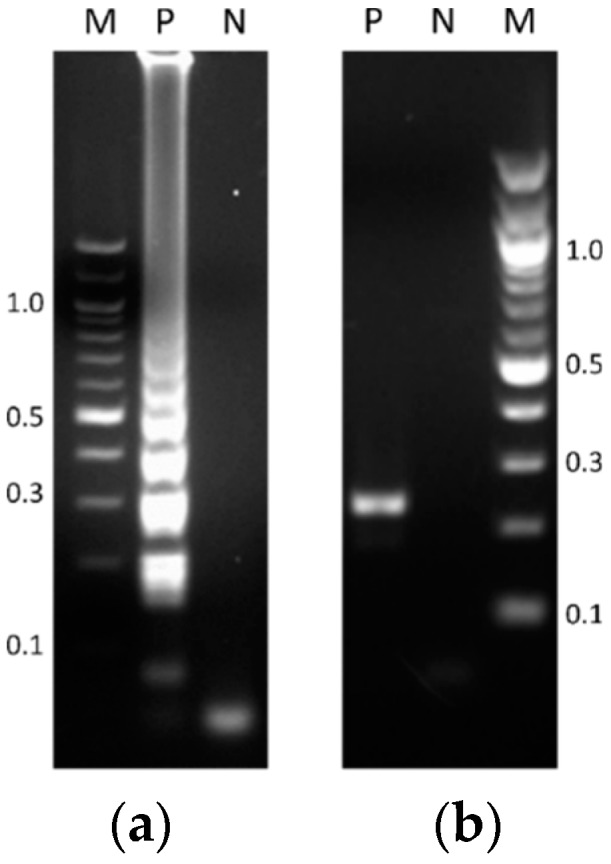
Agarose gel electrophoresis of products obtained by (**a**) the typical loop-mediated isothermal amplification (LAMP) reaction performed in a heating block, and (**b**) the standard endpoint polymerase chain reaction (PCR) assay. Lanes: M, molecular weight (MW) markers (100 bp DNA ladder, New England Biolabs); P, positive control (1 ng of reference DNA); N, negative control (blank reaction).

Reliable isothermal amplification reactions validated the reproducibility of the TB-specific LAMP assay as performed in a heating block. Using genetic material of the *M. tuberculosis* H37Rv strain as the template (reference DNA), positive reactions produced the typical ladder-like pattern (Figure 1a). Likewise, endpoint PCR reactions yielded the expected 233 bp sized product, (Figure 1b).

### 3.2. Detection of TB in Sputum Specimens by the DNAzol–LAMP Procedure

**Figure 2:**
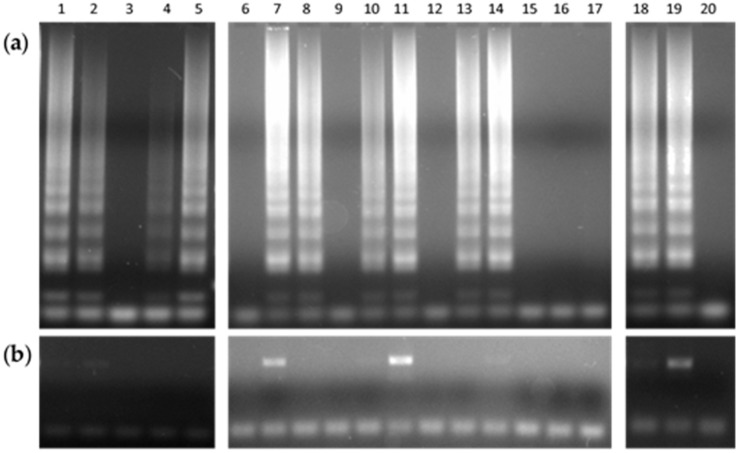
Agarose gel electrophoresis of products obtained by (**a**) the typical LAMP reaction performed in a heating block, and (**b**) the standard endpoint PCR assay, from 20 sputum samples.

Patients who attended the TB Clinic of the GHT, suspected of having TB disease, provided the sputum specimens prior to starting any drug therapy. Immediately, BBL MycoPrep protocol fulfilled the proper digestion and decontamination of sputum samples. Afterwards, smear microscopy and bacterial culture completed the routine mycobacteriology analysis. Then, of the smear-positive pool, 20 samples served as the biological analytes for testing the DNAzol–LAMP procedure (Figure 2).

As observed (Table 1), 60% samples tested positive for both LAMP and bacterial culture, but only 20% rendered the same outcome for PCR.

Although the comparison LAMP/PCR revealed good values by kappa (*κ*) statistics (Table 2), the LAMP/culture showed superior agreement.

Together, these results suggest that LAMP (performed in a heating block) provided technical settings that were suitable for the isothermal amplification of mycobacterial DNA. Furthermore, the DNAzol–LAMP procedure exhibited reliable conditions for TB detection in sputum specimens, as demonstrated by the indicators of diagnostic test performance (Table 3).

As a final remark, it is fair notice that DNAzol–LAMP showed the ability of detection in sputum samples with apparent low-titers of live bacteria. Acknowledging that cell viability decreases with the storage time, it is reasonable to propose a thorough examination of such an advantage, as it represents an attractive improvement in the production of accurate results, and a significant reduction in the unpleasant practice of repeating the test or requesting a fresh sample.

## 4. Conclusions

Here, we report a simple DNAzol–LAMP procedure for the detection of *M. tuberculosis* in sputum specimens. We demonstrate the technical feasibility of performing the isothermal amplification in a heating block. By the reliable amplification of mycobacterial DNA, we confirm that the genetic material extracted from smear-positive sputum samples is a suitable template for the LAMP assay.

## Figures and Tables

**Table 1:** Results of molecular and culture testing for detection of tuberculosis (TB) in smear-positive sputum samples.

Sample	LAMP	PCR	Culture
01	+	-	+
02	+	+	+
03	-	-	+
04	+	-	+
05	+	-	-
06	-	-	-
07	+	+	+
08	+	-	-
09	-	-	-
10	+	-	+
11	+	+	+
12	-	-	-
13	+	-	-
14	+	-	+
15	-	-	-
16	-	-	-
17	-	-	-
18	+	-	-
19	+	+	+
20	-	-	-

**Table 2:** Results of agreement between LAMP assay and others (i.e., PCR or culture) for detection of TB in smear-positive sputum samples.

LAMP	PCR		Culture	
	Positive	Negative	Positive	Negative
Positive	4	8	8	4
Negative	0	8	1	7
Agreements	60%		75%	
κ-Coefficient	0.29 ± 0.14		0.51 ± 0.18	
Strength	Fair		Moderate	

**Table 3:** Diagnostic performance of the TB-specific LAMP assay performed in a heating block.

Sensitivity	88.9%
Specificity	63.6%
PPV	66.7%
NPV	87.5%

PPV, positive predictive value; NPV, negative predictive value.

## Limitations

The reliability of outcome justifies the application of DNAzol–LAMP as a simple procedure for the routine diagnosis of TB in sputum samples. However, this notion must be assessed through a large-scale study to meet the sensitivity and specificity required clinical purposes.
